# Electrochemical micro-aptasensors for exosome detection based on hybridization chain reaction amplification

**DOI:** 10.1038/s41378-021-00293-8

**Published:** 2021-08-17

**Authors:** Wenfen Zhang, Zhenhua Tian, Shujie Yang, Joseph Rich, Shuaiguo Zhao, Mikael Klingeborn, Po-Hsun Huang, Zhishang Li, Alexander Stout, Quinn Murphy, Edward Patz, Shusheng Zhang, Guozhen Liu, Tony Jun Huang

**Affiliations:** 1 College of Chemistry, Zhengzhou University, Zhengzhou, Henan 450001 People’s Republic of China; 2 Department of Mechanical Engineering and Materials Science, Duke University, Durham, NC 27708 USA; 3 Department of Biomedical Engineering, Duke University, Durham, NC 27708 USA; 4 Department of Ophthalmology, Duke University, Durham, NC 27708 USA; 5 College of Biosystems Engineering and Food Science, Zhejiang University, Hangzhou, Zhejiang 310058 People’s Republic of China; 6 Department of Radiology, Duke University, Durham, NC 27708 USA; 7 School of Life and Health Sciences, The Chinese University of Hong Kong, Shenzhen, Guangdong 518172 China

**Keywords:** Chemistry, Electrical and electronic engineering, Biosensors

## Abstract

Exosomes are cell-derived nanovesicles that have recently gained popularity as potential biomarkers in liquid biopsies due to the large amounts of molecular cargo they carry, such as nucleic acids and proteins. However, most existing exosome-based analytical sensing methods struggle to achieve high sensitivity and high selectivity simultaneously. In this work, we present an electrochemical micro-aptasensor for the highly sensitive detection of exosomes by integrating a micropatterned electrochemical aptasensor and a hybridization chain reaction (HCR) signal amplification method. Specifically, exosomes are enriched on CD63 aptamer-functionalized electrodes and then recognized by HCR products with avidin-horseradish peroxidase (HRP) attached using EpCAM aptamers as bridges. Subsequently, the current signal that is generated through the enzyme reaction between the HRP enzyme and 3,3’,5,5’-tetramethylbenzidine (TMB)/H_2_O_2_ directly correlates to the amount of bound HRP on the HCR products and thus to the number of target exosomes. By introducing anti-EpCAM aptamers, micro-aptasensors can detect cancerous exosomes with high specificity. Due to the micropatterned electrodes and HCR dual-amplification strategy, the micro-aptasensors achieve a linear detection response for a wide range of exosome concentrations from 2.5×10^3^ to 1×10^7^ exosomes/mL, with a detection limit of 5×10^2^ exosomes/mL. Moreover, our method successfully detects lung cancer exosomes in serum samples of early-stage and late-stage lung cancer patients, showcasing the great potential for early cancer diagnosis.

## Introduction

Exosomes, nanosized vesicles with diameters of 30–150 nm, are released by cells during the fusion of multivesicular endosomes with plasma membranes^1,2^. Exosomes transfer RNA and proteins to recipient cells, thereby facilitating cell-to-cell communication. Recent studies^3–5^ have demonstrated that the expression profiles of exosomal nucleic acids and proteins are altered in many diseases, including cancer, cardiovascular illnesses, infectious diseases, diabetes, neurodegenerative diseases, and depression, demonstrating their promise as a noninvasive biomarker for early detection and diagnosis. For example, many studies show that exosomes secreted by cancer cells carry abundant information about the cells in the form of various biological molecules, such as proteins, lipids, RNA, and DNA fragments^6–9^. Some recent studies present evidence that exosomes secreted by cancer cells could contribute to the pathological process and even promote tumor growth, migration, invasion, and chemoresistance^10–14^. In this regard, exosomes could be an excellent biomarker for early cancer diagnosis, and the concentration of cancer-specific exosomes could be used to determine the corresponding stage of cancer^15–17^.

Great efforts have been devoted to developing methods for exosome analysis and detection based on fluorescence^18,19^, colorimetry^20,21^, electrochemistry^22–27^, surface-enhanced Raman scattering (SERS)^28,29^, surface plasmon resonance (SPR)^30,31^, photoelectrochemical (PEC)^32^, and mass spectrometry^33^. In particular, various electrochemical biosensors have been developed for exosome detection using antibodies as recognition molecules^34–37^. One attractive alternative to antibodies is aptamers (i.e., short ssDNA and RNA molecules). Aptamers bind to desired targets with excellent specificity and exhibit merits such as high stability, easy synthesis, and low cost^10,38–40^. Several aptamer-based biosensors have been developed for the determination of exosomes. Li et al. developed an aptasensor based on a hemin/G-quadruplex-assisted signal amplification strategy to perform electrochemical detection of gastric cancer exosomes^24^. Tan et al. developed an aptamer-based nanotetrahedron-assisted biosensor to capture and detect hepatocellular exosomes^41^. However, most existing aptamer-based methods are nonspecific target capture methods, which are based on nonspecific markers^42^ or chemical reactions^27^. These nonspecific capture methods make it difficult to distinguish between tumor and nontumor exosomes. Therefore, it is highly desirable to develop new aptasensors to overcome the limitations of previous methods for the detection of specific exosomes (e.g., cancerous exosomes).

This study presents sensitive, specific micro-aptasensors for the detection of cancerous exosomes. Our micro-aptasensors fuse the merits of microfabricated electrodes, electrochemical analysis, hybridization chain reaction (HCR)-based signal amplification, and multiple aptamers. In our protocol, long HCR products can attach multiple avidin-horseradish peroxidases (HRPs) for efficient signal amplification for the sensitive detection of exosomes in the sample^43–46^. In addition, by introducing anti-EpCAM aptamers, our micro-aptasensors enable the detection of EpCAM-positive exosomes with high specificity. Based on these mechanisms, our micro-aptasensors achieve the sensitive, specific, and reliable detection of exosomes secreted by cancer cells and quantitative evaluation of the exosome concentration. Proof-of-concept experiments have been performed to detect exosomes secreted by multiple cell lines as well as exosomes isolated from patient serum samples. The performance of our micro-aptasensors was successfully validated by detecting lung cancer exosomes in serum samples from early- and late-stage lung cancer patients. We believe our developed micro-aptasensors have the potential to become a powerful exosome-based disease diagnosis platform.

## Results and discussion

### Mechanism of the electrochemical micro-aptasensors

**Fig. 1:**
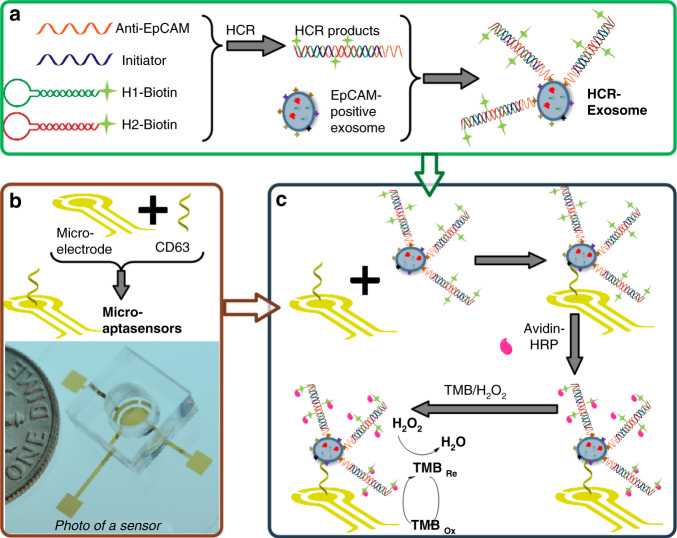
Schematics illustrating the mechanism of electrochemical micro-aptasensors based on hybridization chain reaction (HCR). **a** Procedures for preparing biotin-labeled HCR exosomes. **b, top** Modification of microelectrodes with CD63. **b, bottom** A photo of a fabricated electrochemical micro-aptasensor with CD63-modified electrodes and a microfluidic chamber. **c** The working mechanism of electrochemical micro-aptasensors.

As shown in Fig. 1, our micro-aptasensor is composed of microelectrodes modified with CD63 aptamers and a microfluidic chamber. The details of the sensor fabrication and modification procedures can be found in the Experimental Section (Fig. S1). The mechanism of our electrochemical micro-aptasensor is illustrated in Fig. 1. When the HCR-exosome samples were added to the microfluidic chamber, the CD63 aptamers on the microelectrodes captured the exosomes, and the anti-EpCAM aptamers enabled HCR. Biotins labeled with H1 and H2 were used to bind with the anti-EpCAM aptamers and further produce long chains with multiple biotins. This process allows both signal amplification and the specific recognition of cancerous exosomes. As shown in Fig. 1c, a sandwich structure composed of CD63, exosomes, and anti-EpCAM-embedded HCR products was formed. For the electrochemical analysis, the current signals, which were generated through a reaction between the HRP enzyme and TMB/H_2_O_2_, were monitored by an electrochemical analyzer through microelectrodes. Details of the electrochemical analysis procedures are given in the Experimental Section. Due to the specific recognition between avidin-HRP and biotin, the current signal generated through the enzyme reaction is directly related to the amount of HRP on the HCR products and the exosome concentration.

### Device optimization

**Fig. 2:**
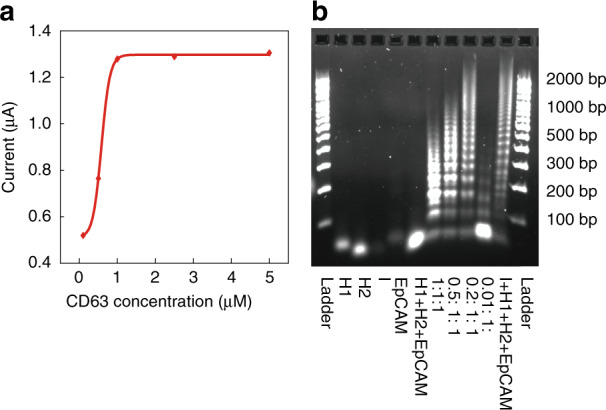
Optimization of aptamer concentrations. **a** Measured current values for different CD63 concentrations that are used for the modification of microelectrodes. **b** Gel electrophoresis results for investigating the influence of initiator (I) concentrations on HCR. Lanes 1 and 12 are for DNA ladder markers. Lanes 2-5 are H1, H2, I, EpCAM, and a mixture of H1, H2, and EpCAM, respectively. Lanes 7-10 are for four different initiator concentrations 1.0, 0.5, 0.2, and 0.01 μM in solutions with 1 μM H1 and 1 μM H2, respectively. Lane 11 is for the mixture of I, H1, H2, and EpCAM.

To determine the optimal concentration of CD63 aptamers for the modification of our micro-aptasensors, we performed experiments with five different concentrations (0.1, 0.5, 1.0, 2.5, and 5.0 µM) of CD63. As shown in Fig. 2a, as the CD63 concentration was increased from 0.1 to 1.0 µM, the measured current increased. As the CD63 concentration was further increased from 1.0 to 5.0 µM, the current remained nearly constant. Therefore, a 1.0 µM concentration of CD63 was selected for electrode modification in this study.

To maximize the signal amplification effect with longer HCR products, we optimized the concentration of initiator aptamers for HCR according to a previous method^47^. As shown in Fig. 2b, when the concentration of initiator aptamers (lane 9) was 0.2 μM, the HCR product length was longer than the lengths for other concentrations. Moreover, the length of the HCR product with the anti-EpCAM aptamer (lane 11) increased slightly compared to the length of the HCR product (lane 9) without the anti-EpCAM aptamer. This result showed that the anti-EpCAM aptamer can successfully hybridize with H1, H2, and I. The longest product was over 2,000 base pairs. This showed that the HCR method is efficient for signal amplification.

### Sensitivity and detection limit of the electrochemical micro-aptasensors

**Fig. 3:**
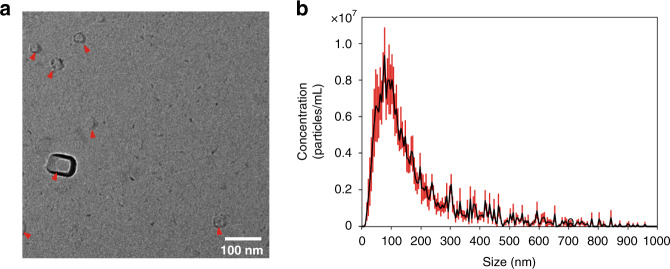
Characterization of exosomes isolated from cell culture media. **a** A TEM image shows the morphologies of isolated cup-shaped exosomes. **b** The NTA characterization result shows the size distribution of isolated exosomes.

To characterize the sensitivity and detection limit of our micro-aptasensors, exosomes isolated from MCF-7 cell culture medium were used^48^. The isolated exosomes were characterized using transmission electron microscopy (TEM) and nanoparticle tracking analysis (NTA). As shown in Fig. 3a, the isolated exosomes exhibited a typical cup-shaped appearance. The size distribution of exosomes ranged from 30 to 200 nm, and the concentration was 1.91×10^8^ exosomes/mL, as shown in Fig. 3b.

**Fig. 4:**
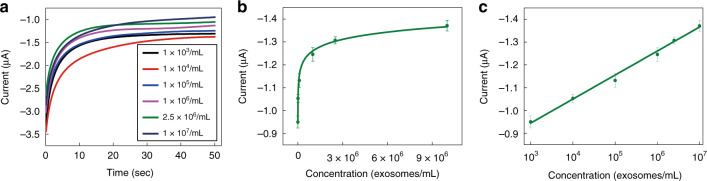
Electrochemical detection of exosomes with the electrochemical micro-aptasensor. **a** Multiple current-time curves for different concentrations of exosomes. **b** Concentration-dependent signal amplification of micro-aptasensors. **c** The calibration curve for our micro-aptasensors. Error bars represent the relative standard deviation of measurements (RSD ≤ 3.0%).

The current responses to MCF-7-derived exosomes at different concentrations (1×10^3^, 1×10^4^, 1×10^5^, 1×10^6^, 2.5×10^6^ and 1.0×10^7^ exosomes/mL) were measured. As shown in Fig. 4a and b, as the concentrations of exosomes were increased from 2.5×10^3^ to 2.5×10^6^ exosomes/mL, the measured current values significantly increased. As the concentrations were further increased from 2.5×10^6^ to 1.0×10^7^ exosomes/mL, the current value increased only slightly because the analyte-capture capability of our micro-aptasensors gradually achieved saturation. Through a data regression step, a linear relationship (shown in Fig. 4c) was found between the current value and the logarithm of the exosome concentration, as expressed by the equation *I*_*c*_ = −0.08305 log *c* −0.8012 (*R*^2^=0.9910), where *I*_*c*_ represents the signal intensity, and *c* represents the exosome concentration. As shown in Table S1 in the Supporting Information, our aptasensor exhibits high sensitivity with a detection limit of 0.5 exosomes/μL, which is significantly lower than the limits of most published methods^23–27,49–51^. The high sensitivity of our method is related to the high electron transfer efficiency of our microelectrodes and the HCR amplification strategy.

### Specificity and reliability of the electrochemical micro-aptasensors

**Fig. 5:**
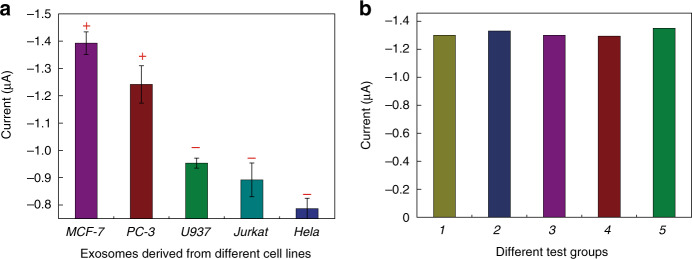
Exosome detection results for characterizing the specificity and reliability of the electrochemical micro-aptasensors. **a** Comparison among measured current values for exosomes isolated from the culture media of EpCAM-positive (MCF-7 and PC-3) and EpCAM-negative (U937, Jurkat, and HeLa) cell lines. +: EpCAM-positive; −: EpCAM-negative. **b** Comparison among measured current values for 5 different electrochemical micro-aptasensors operated under the same conditions.

To investigate the specificity of our micro-aptasensors, experiments were performed with exosomes derived from multiple cell lines, including EpCAM-positive cell lines (MCF-7 and PC3) and EpCAM-negative cell lines (Jurkat, HeLa, and U937). As shown in Fig. 5a, only exosomes derived from EpCAM-positive cell lines gave high current responses with our micro-aptasensors. For EpCAM-negative cell lines, the measured current values were much lower. These results proved that our method has high specificity for EpCAM-positive exosomes.

To investigate the reliability of our method, micro-aptasensors were fabricated and used under the same conditions to detect EpCAM-positive exosomes at a concentration of 2.0×10^6^ exosomes/mL. As shown in Fig. 5b, the current values measured from five different sensors had a small relative standard deviation of 1.86%. This relatively small standard deviation showed that our method gives consistent detection results among different devices.

### Detection of cancerous exosomes isolated from human serum samples

**Fig. 6:**
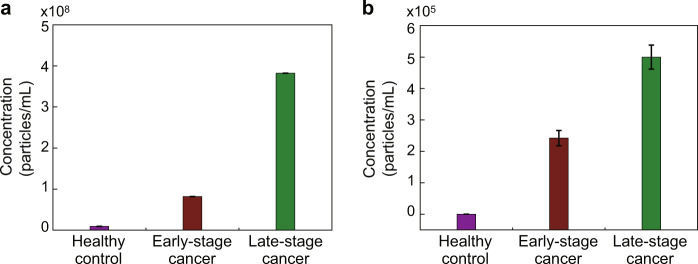
Results for detecting exosomes in serum samples of healthy individuals and early-stage and late-stage lung cancer patients. Detection results using **a** NTA and **b** our electrochemical micro-aptasensors.

To assess their potential in preclinical/clinical applications, the developed micro-aptasensors were used for detecting exosomes in human serum samples. Exosomes were isolated from the serum samples of 2 healthy individuals as well as 2 early-stage and 2 late-stage lung cancer patients. Figure 6a and b show the exosome detection results by NTA and our method, respectively. The exosome amounts in the early-stage and late-stage lung cancer samples were significantly higher than the exosome amounts in the healthy patient samples. The exosome amount in the late-stage lung cancer sample was significantly higher than the exosome amounts in both the early-stage lung cancer and the healthy patient samples. Compared to the NTA analysis (Fig. 6a), lower exosome concentrations were detected by our method (Fig. 6b). This difference is likely because the NTA-based method detects not only cancerous exosomes but also noncancerous exosomes^51^^,^ while our method detects only cancerous exosomes. These experimental results demonstrated that our electrochemical micro-aptasensors can not only detect cancerous exosomes in patient samples and distinguish them from noncancerous exosomes but also can differentiate samples from early-stage lung cancer patients from those from late-stage patients. In this regard, our electrochemical micro-aptasensors have great potential for early cancer detection and the identification of the corresponding stage of cancer.

## Conclusions

We have established sensitive, specific, and reliable electrochemical micro-aptasensors for the detection of exosomes by taking advantage of both electrochemical analysis and HCR-based signal amplification. With the introduction of anti-EpCAM aptamers, our developed micro-aptasensors can achieve high specificity for the detection of EpCAM-positive cancerous exosomes. Moreover, the microelectrodes with the HCR-based signal amplification approach greatly improve the detection sensitivity. In addition to exosomes from cell culture medium, our micro-aptasensors have been successfully applied for the detection of cancerous exosomes in serum samples of early-stage and late-stage lung cancer patients, shedding new light on early cancer diagnosis.

## Experimental section

### Exosome isolation

Two EpCAM-positive cell lines (MCF-7 and PC3) and three EpCAM-negative cell lines (Jurkat, HeLa, and U937) were cultured to secrete exosomes. MCF-7 and HeLa cells were cultured in DMEM (Gibco, Life Technologies, MA, USA) with 5% exosome-depleted fetal bovine serum (Gibco, Thermo Fisher, MA, USA) and 1% penicillin streptomycin (Gibco, Life Technologies, MA, USA). PC3, Jurkat, and U937 cells were cultured in RPMI-1640 (Gibco, Life Technologies, MA, USA) with 5% exosome-depleted fetal bovine serum and 1% penicillin streptomycin. To isolate exosomes from the culture medium, the medium was centrifuged at 2000×*g* for 10 min at room temperature. The supernatant was then centrifuged at 10,000 *g* for 30 min in an ultracentrifuge (Optima XE, Beckman, CA, USA) to remove large microvesicles, such as ectosomes and oncosomes. The supernatant from this centrifugation was further centrifuged at 100,000 *g* for 2 h. After all the centrifugation steps, the collected pellet contained only exosomes and other small particles, such as lipoproteins. The collected pellet was resuspended in phosphate-buffered saline (PBS, Invitrogen, CA, USA) for further analysis.

To isolate exosomes from the serum samples of lung cancer patients, exosome isolation reagent (Thermo Fisher, MA, USA) was used following the manufacturer’s instructions starting with 200 μL of each serum sample. All serum samples were obtained from the Department of Ophthalmology at Duke University and represented 2 healthy individuals (H), 2 early-stage lung cancer patients, and 2 late-stage of lung cancer patients (P). The samples used for this study were approved by the Duke University Health System Institutional Review Board, and all subjects gave written informed consent (Protocol ID: Pro00012914).

### Fabrication and modification of micro-aptasensors

As shown in Fig. 1, the micro-aptasensor is composed of microelectrodes modified with CD63 aptamers and a microfluidic chamber. The microelectrodes were fabricated through standard lithography^23^, E-beam evaporation, and lift-off procedures on a 500 μm thick fused silica substrate (University wafer, MA, USA). During the E-beam evaporation process, a double-metal layer with Ti (10 nm, bottom) and Au (400 nm, top) was deposited on the substrate to form microelectrodes. After fabrication, the microelectrodes were modified. The CD63 aptamer stock solution (100 μM) was mixed with 10 mM tris(2-carboxyethyl) phosphine (TCEP, Sigma Aldrich, MO, USA) for 1 h to cleave the disulfide bonds, and then 4-(2-hydroxyethyl)-1-piperazineethanesulfonic acid (HEPES, Sigma Aldrich, MO, USA) buffer was used to further dilute the CD63 aptamers to 1 μM. Then, 30 μL of the prepared solution of CD63 aptamers was placed on the microelectrodes for overnight incubation. After incubation, the microelectrodes were washed with deionized (DI) water. Next, 30 μL of 1 mM 6-mercapto-1-hexanol (MCH, Sigma Aldrich, MO, USA) was added to the microelectrodes for 15 min. To remove the excess MCH, the microelectrodes were washed thoroughly with DI water. In the experiments, a polydimethylsiloxane (PDMS) chamber was used to prevent lateral spreading of the liquid sample. Briefly, PDMS was first prepared through the standard mold-replication method^52^ and then cut to the appropriate size and drilled. Finally, the open PDMS chamber was bonded to the glass substrate to expose the patterned electrodes and prevent the added solution from leaking.

### Hybridization chain reaction (HCR)

To perform HCR, 2 μM H1 and 2 μM H2 (Table 1) were prepared by diluting stock solutions with Tris buffer containing 50 mM MgCl_2_ (TM) buffer (20 mM, pH 8.0). Then, the prepared H1 and H2 solutions were heated to 95 °C for 10 min and immediately cooled to room temperature using ice. A solution of 10 μM anti-EpCAM aptamers and 10 μM initiator (I) strands was prepared. The above prepared solutions were then mixed to obtain the prepared hybridization mixture containing anti-EpCAM aptamers I, H1, and H2 at concentrations of 0.2, 0.2, 1, and 1 μM, respectively. The prepared hybridization mixture was kept at 37 °C overnight to obtain the HCR products.

**Table 1:** DNA sequences used in this study.

	DNA sequence (5′–3′)
**Anti-EpCAM**	CACTACAGAGGTTGCGTCTGTCCCACGTTGTCATGGGGGGTTGGCCTGTTTGCAAAGCTTACGGCATACGT
**I**	CTAGAGCACAATCACAGGAGCCAGTTTACGTATGCCGTAAGCTTTGC
**H1**	biotin-TTTTTTTTTTCTGGCTCCTGTGATTGTGCTCTAGTTTACATCGCTAGAGCACAATCACAGG
**H2**	biotin-TTTTCTAGAGCACAATCACAGGAGCCAGTTACCTGTGATTGTGCTCTAGCGATG
**CD63**	CACCCCACCTCGCTCCCGTGACACTAATGCTA/iSpC3//3ThioMC3-D-30

### Exosome detection through electrochemical analysis

For exosome detection, HCR-exosomes were prepared by adding approximately 1 million exosomes to 1 mL of a solution of HCR products and then shaken at 4 °C for 1 h (Fig. S2A in the Supporting Information). Next, 40 μL of HCR-exosome solution was added to the CD63-modified microelectrodes and incubated for 40 min at room temperature (Fig. S2B in the Supporting Information). Then, 2.5 μL of 10 μm/mL avidin−HRP (horseradish peroxidase coupled with avidin, Sigma Aldrich, MO, USA) was added to the microelectrodes. After 15 min at room temperature, the microelectrodes were rinsed to prepare for electrochemical analysis. An electrochemical analyzer (CHI 800B, TX, USA) was used for the analysis. 3,3′,5,5′-Tetramethylbenzidine (TMB) substrate (Neogen K-blue low-activity substrate, Shanghai, China) in H_2_O_2_ was used as the reaction solution^38^. The electroreduction current was measured for 50 s with an applied voltage of 100 mV after the HRP redox reaction reached steady state.

### Characterization methods

#### Transmission electron microscopy (TEM) characterization

TEM was conducted according to the protocols outlined in the Supporting Information and used for morphological characterization of the exosomes.

#### Nanoparticle tracking analysis (NTA)

The size distribution and concentration of the isolated exosomes were characterized with an NTA (Nanosight LM10, Malvern, USA) system.

#### Electrophoresis

A 40 mL 1% agarose gel containing 4 μL of SYBR safe DNA stain (Thermo Fisher Scientific, MA, USA) was prepared with TAE buffer (40 mM Tris-acetate and 1 mM EDTA, pH 8.0). Gel electrophoresis of HCR products was performed at 100 V for 40 min using a Bio-Rad PowerPacTM Basic Electrophoresis Analyzer (Bio-Rad, CA, USA) and imaged using a Bio-Rad ChemDoc Touch Imaging System (Bio-Rad, CA, USA).

#### DNA sequences

All the DNA sequences used in this study were synthesized by Sangon Biotech (Shanghai, China). Their sequences are given in Table 1.

## Supplementary information

Supporting information-marked Up
