# Supplementary material for: Electrochemical micro-aptasensors for exosome detection based on hybridization chain reaction amplification
Source: Microsyst Nanoeng. 2021 Aug 17;7:63. doi: 10.1038/s41378-021-00293-8 (PMC8433316; doi:10.1038/s41378-021-00293-8)
Supplement: Supplementary file 1 — Supporting information-marked Up [file 41378_2021_293_MOESM1_ESM.docx]

Supporting Information

**Electrochemical micro-aptasensors for exosome detection based on hybridization chain reaction amplification**

*Wenfen Zhang^1,2^, Zhenhua Tian^2^, Shujie Yang^2^, Joseph Rich^3^, Shuaiguo Zhao^2^, Mikael Klingeborn^4^, Po-Hsun Huang^2^, Zhishang Li^5^, Alexander Stout^2^, Quinn Murphy^2^, Edward Patz^6^, Shusheng Zhang^1*^, Guozhen Liu^7^, Tony Jun Huang^2*^*

^1^ College of Chemistry, Zhengzhou University, Zhengzhou, Henan 450001, P. R. China;

^2^ Department of Mechanical Engineering and Materials Science, Duke University, Durham, NC 27708, United States

^3^ Department of Biomedical Engineering, Duke University, Durham, NC 27708, United States

^4^ Department of Ophthalmology, Duke University, Durham, NC, United States

^5^ College of Biosystems Engineering and Food Science, Zhejiang University, Hangzhou, Zhejiang 310058, P. R. China

^6^ Department of Radiology, Duke University, Durham, NC, United States

^7^ School of Life and Health Sciences, The Chinese University of Hong Kong, Shenzhen, Guangdong, China, 518172

*Email: [tony.huang@duke.edu](mailto:tony.huang@duke.edu), zsszz@126.com

**Chemicals and materials.** All the cell lines used in this work were purchased from the American Type Culture Collection (USA). Glass wafers used for electrode fabrication were purchased from University wafer (MA, USA). All DNA sequences shown in Table 1 were synthesized and modified by Sangon Biotech (Shanghai, China). TMB substrates (3,3’,5,5’-tetramethylbenzidine; Neogen K-blue low-activity substrate, H_2_O_2_ included) were purchased from Neogen (Shanghai, China). 6-Mercapto-1- hexanol (MCH) and horseradish peroxidase coupled with avidin (Avidin-HRP) were purchased from Sigma Aldrich (MO, USA). The hybridization chain reaction buffer (TM buffer) was Tris buffer (20 mM, pH 8.0) containing 50 mM MgCl_2_ (Sigma Aldrich, MO, USA). The washing buffer was phosphate buffered saline (PBS) solution (10 mM phosphate buffer, 0.14 M NaCl, 2.7 mM KCl, pH 7.4). Milli-Q water (18 MΩ·cm−1 resistivity) was used throughout all experiments. SYBR safe DNA gel stain was purchased from Thermo fisher scientific (MA, USA).

**Instruments.** A CHI 800B electrochemical workstation (Austin, USA) was employed for electrochemical detection, which was performed from -0.4 to 0.4 V at a scan rate of 100 mV/s. And it was also used for amperometric detection, which was obtained at 100 mV within 50 s. A homemade three-electrode system was used (gold electrode was used as the working electrode, reference electrode, and counter electrode) for all the experiments. The exosome isolation was performed on an Optima XE ultracentrifuge (Beckman, California, USA). The exosome content isolated from cell culture medium was detected by NTA (Nanosight, Salisbury, England). A FEI Tecnai G^2^ Twin transmission electron microscope (MA, USA) was used for the morphological characterizations. The gel electrophoresis of the HCR products was performed on a BIO RAD PowerPac^TM^ Basic Electrophoresis Analyser (Bio-Rad, California, USA) and imaged on the Bio-rad ChemDoc Touch Imaging System (Bio-Rad, USA).

**TEM characterization.** Equal quantities of exosome sample were mixed with a 2% aqueous solution of the stain (and adjusted the pH to 7.0 with 1M KOH, if required). Then a drop of this mixture was placed onto the grid and almost all the solution was removed with filter paper after 20 s. Following that, the grid was washed twice with DI water and dried in air.

**Characterization of the electrochemical micro-aptasensor.** The cyclic voltammetry (CV) was carried out to monitor each modification step in 1.0 mM K_3_[Fe(CN)_6_] solution containing 0.1 M KCl. As shown in Fig. S1A, a couple of reversible redox peaks of bare gold showed a peak potential separation (ΔE_p_) at approximately 90 mV. After the self-assembly of CD63 aptamers on the bare Au electrodes, the current intensity exhibited an obvious reduction. This reduction may be due to the negatively charged phosphate backbone of the aptamers that can effectively inhibit the diffusion of [Fe(CN)_6_]- from solution to the electrode surface. This inhibition of diffusion from the aptamers suggests that the CD63 aptamer was successfully modified on the electrode. After reducing the non-specific adsorption with MCH, the current intensity decreased further, attributing to the sealing of the electrode. In addition to this, when the exosomes were captured on the modified electrode, a sharp decrease in current intensity was found. This may be because exosomes can significantly inhibit the diffusion of [Fe(CN)_6_]- to the electrode surface, due to the negatively charged lipid bilayers and proteins on their surface.


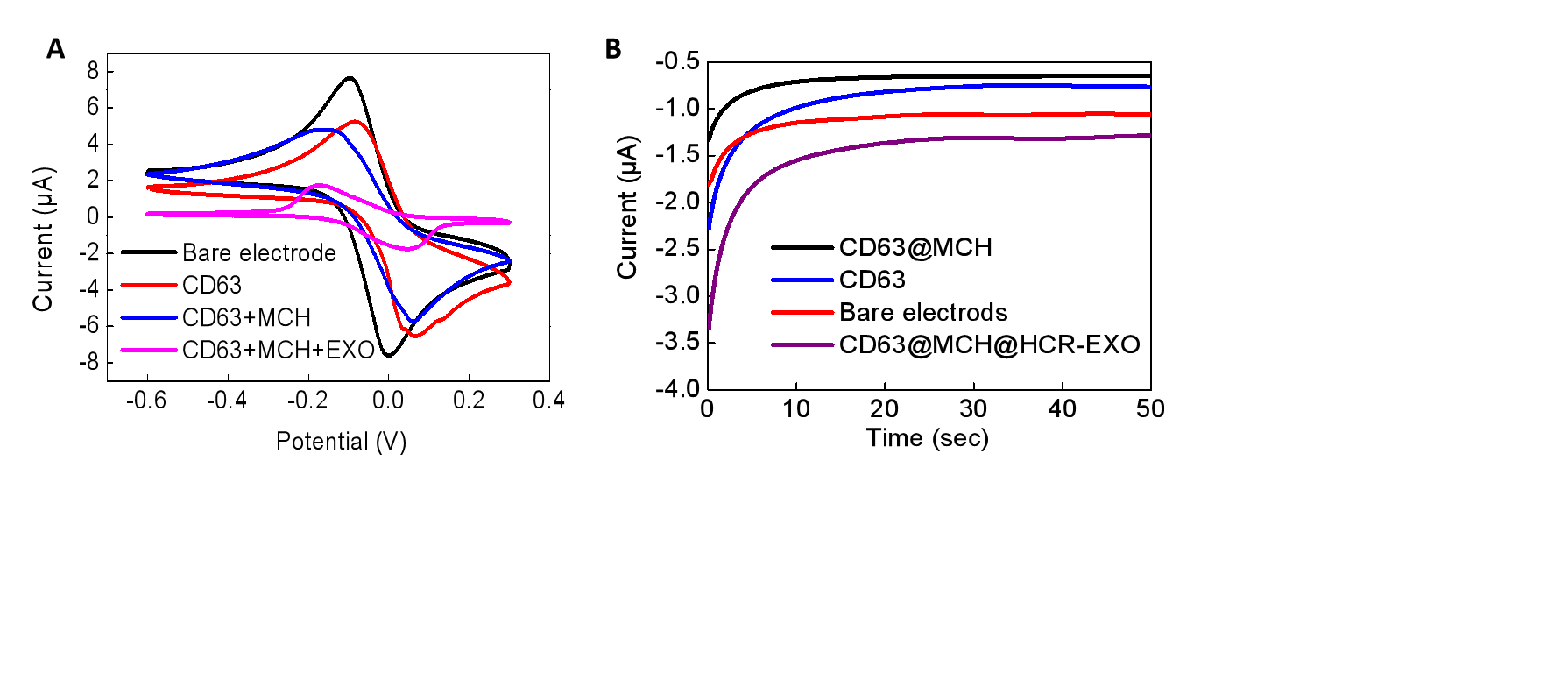


**Fig. S1 Characterization of the bare electrode
and the electrochemical micro-aptasensor.**

**The optimization of the detection condition.** The incubation time between exosomes and HCR products was optimized. As shown in Fig S2A, the current increased with the incubation time from 20 min to 60 min and remained constant as the incubation time increased from 60 min to 80 min. Thus, 60 min was selected for the following experiments. The CD63 capture time of HCR-Exo was also optimized. As shown in Fig S2B, 40 min is enough to get a higher current intensity. Thus, 40 min was selected as the capture time.


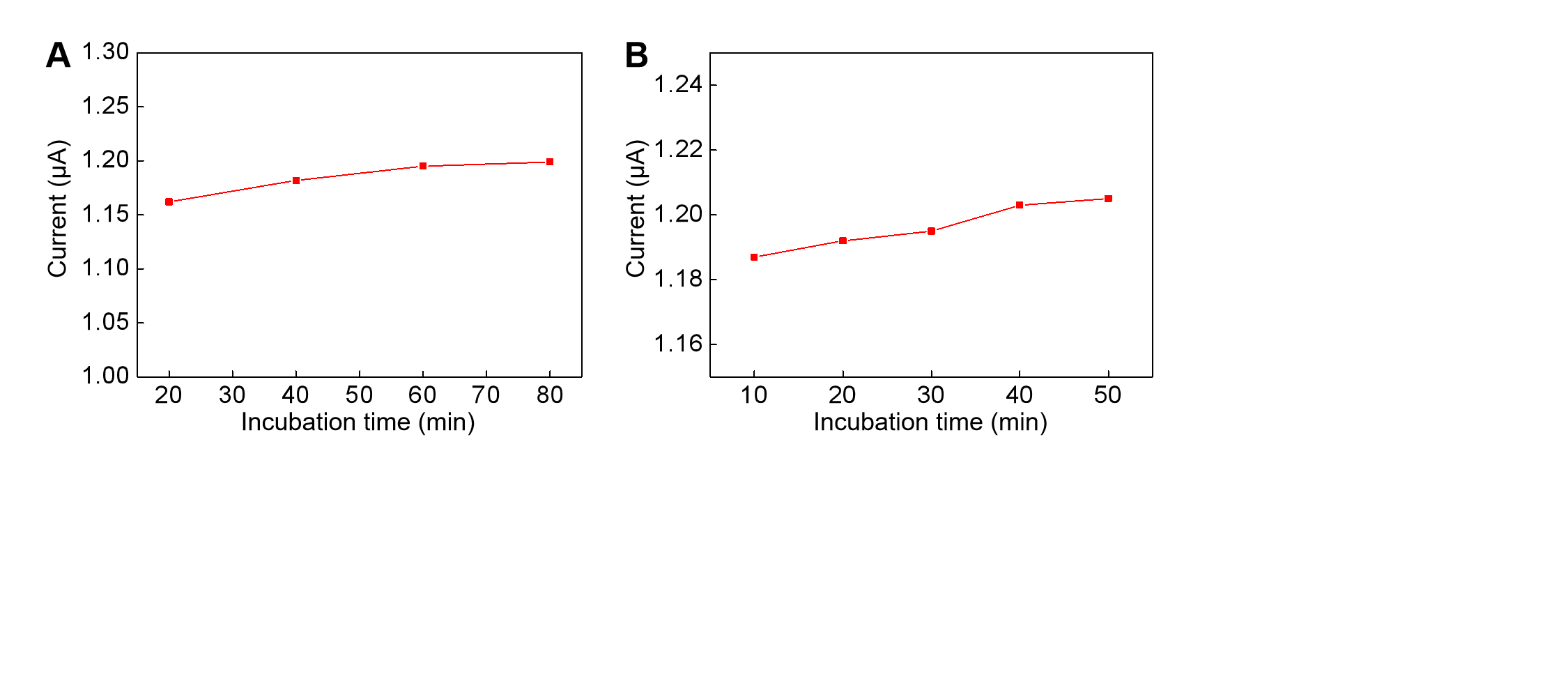


**Fig. S2 The optimization of detection condition.**

**Table S1. Summary of electrochemical biosensors for the detection of exosomes**

| **Specific target** | **Source of exosomes** | **Electrode type** | **Detection method** | **Amplification method** | **Detection limit (particles/mL)** | **Selective detection** | **Reference** |
| --- | --- | --- | --- | --- | --- | --- | --- |
| CD63 | HepG2 cells | Micropatterned gold electrode | Square wave voltammetry (SWV) |  | 1×10^6^ | no | ^23^ |
| Anti-CD63 | Gastric cancer exosome | Conventional three-electrode system | Pulse voltammetry (DPV) | Rolling circle amplification | 9.54 × 10^2^ | Yes | ^24^ |
| Anti-CD63 | HepG2 and MCF-10A cells | Conventional three-electrode system | Square wave voltammetry (SWV) | Catalytic molecule machine | 1.72×10^4^ | No | ^26^ |
| CD63 aptamer | MCF-7 cells | Glassy carbon electrode for | Pulse voltammetry (DPV) | Hybridization and chain reaction | 9.6 ×10^4^ | No | ^27^ |
| CD63 | MCF-7 and HL-7702 cells | ITO electrodes | Pulse voltammetry (DPV) | Exonuclease III-assisted recycling amplification | 1.2 ×10^4^ | No | ^51^ |
| CD63 and EpCAM aptamer | MCF-7, HeLa, and HEK-293T cells | Au electrode | Pulse voltammetry (DPV) | Three-dimensional DNA walker | 1.3 × 10^4^ | Yes | ^52^ |
| CD63 aptamer | MCF-7 cells | Indium tin oxide (ITO) | Square wave voltammetry (SWV) | Functional hybrid thin-film | 100 particles | No | ^53^ |
| PSMA (prostate-specific membrane antigen) aptamer | LNCaP cells | Conventional three-electrode system | Pulse voltammetry (DPV) | Cyclic enzymatic amplification | 7.0×10^4^ | Yes | ^54^ |
| CD63 and EpCAM | MCF7 | Micropatterned gold electrode | Current-time curves | Hybridization and chain reaction | 5×10^2^ | Yes | This work |
